# Characterization of the Composition Variation of Healthy Human Gut Microbiome in Correlation with Antibiotic Usage and Yogurt Consumption

**DOI:** 10.3390/antibiotics11121827

**Published:** 2022-12-16

**Authors:** Shaofei Yan, Xiaofan Zhang, Xiaofang Jia, Jiguo Zhang, Xiaomin Han, Chang Su, Jianyun Zhao, Wanglong Gou, Jin Xu, Bing Zhang

**Affiliations:** 1 NHC Key Laboratory of Food Safety Risk Assessment, China National Center for Food Safety Risk Assessment, Beijing 100021, China; 2 National Institute for Nutrition and Health, Chinese Center for Disease Control and Prevention, Key Laboratory of Trace Element Nutrition, National Health Commission of the People’s Republic of China, Beijing 100050, China; 3 Key Laboratory of Growth Regulation and Translational Research of Zhejiang Province, School of Life Sciences, Westlake University, Hangzhou 310024, China

**Keywords:** gut microbiota, antibiotic usage, yogurt consumption, metagenomics

## Abstract

Antibiotic usage and yogurt consumption are the major interventions for gut microbiota, yet their shared characteristics and disparities in healthy human gut microbiome remain unclear. This study aimed to decipher the composition changes among healthy humans, comparing antibiotic usage and yogurt consumption. The relative bacterial abundances of 1113 fecal samples were collected from an ongoing, population-based longitudinal cohort study in China that covered lifestyle, diet, disease status and physical measurements, and biological indicators of participants were obtained by the sequencing of 16S rRNA. The samples were divided into three groups, which were antibiotic users (122), yogurt consumers (497) and controls (494), where data visualization, alpha diversity, beta diversity and LEfSe analysis were conducted. At the family level, the relative abundances of *Streptococcaceae*, *Enterobacteriaceae* and *Enterococcaceae* families in antibiotic users increased almost 50%, 70% and 200%, respectively, while yogurt consumption also increased relative abundances of *Streptococcaceae* and *Enterococcaceae*, but not *Enterobacteriaceae*. Alpha diversity analyses suggested that the microbiome of the antibiotic usage and yogurt consumption groups exhibited an alpha diversity lower than that of the control. LEfSe analysis showed that, at the family level, the number of biomarkers in the yogurt consumption and antibiotic usage group were respectively 5 and 7, lower than that of the control (13). This study demonstrated the importance in considering the potential assistance of yogurt consumption on ARG gene transfer from commensal bacteria to pathogens in the human gut, which may pose a risk for human health. Antibiotic usage and yogurt consumption share more identical changes on healthy human gut flora than disparities. Therefore, in order to understand the potential risks of antibiotic usage and yogurt consumption on antibiotic resistance transmission in human gut microbiota, further research needs to be undertaken.

## 1. Introduction

The human gut microbiota comprises the microorganisms that live in the human gut, including bacteria, archaea, fungi, and viruses, from which bacteria are dominant [1]. The functions of gut microbiota are resisting pathogens, maintaining the intestinal epithelium, metabolizing dietary and pharmaceutical compounds, controlling immune function, and so on [2,3]. There are four dominant bacterial phyla in the composition of human gut microbiota: *Bacillota*, *Bacteroidota*, *Actinomycetota*, and *Pseudomonadota* [4]. *Bacteroides*, *Clostridium*, *Faecalibacterium*, *Eubacterium*, *Ruminococcus*, *Peptococcus*, *Peptostreptococcus*, and *Bifidobacterium* are the dominant genera that are found to inhabit the human gut [1]. The composition of human gut microbiota responds to a variety of factors and changes all the time [5]. It has been reported by a human-cohort-based analysis that dynamic change in the gut ecosystem correlates strongly with complex interactions such as host lifestyle, dietary, ecological and other factors [4,6].

Antibiotic resistance is a global concern. Some bacteria in the gut are naturally resistant to certain antibiotics, while other commensal bacteria may acquire resistance genes from fellow resistant bacteria through horizontal gene transfer (HGT), including conjugation, transduction and transformation [7]. The human gut micobiota is directly affected by the clinical use of antibiotics [8]. Antibiotic usage can disrupt the ability of gut micobiota to inhibit pathogen growth, due to the reduction of native bacterial species, therefore causing antibiotic diarrhea [9]. Antibiotic usage may also enhance horizontal AMR gene transfer from commensal bacteria to pathogens in gut microbiota, through which antibiotic resistant pathogens that are difficult to treat with common antibiotics are created [10,11]. Probiotics are microorganisms that are beneficial to health when supplemented as part of the human diet [12]. It has been reported that the consumption of probiotics containing *Lactobacillus* species might help prevent antibiotic-associated diarrhea [13].

Diet may influence gut resistome in healthy humans. Researchers found that subjects with a diverse, fiber-rich diet had a lower abundance of ARGs in their gut, through changing the composition of gut microbiota to harbor more antibiotic resistance genes [14]. As a food type in the human diet that contains probiotics *Lactobacillus delbrueckii* subsp. *bulgaricus* and *Streptococcus thermophilus* bacteria, yogurt consumption is gradually increasing around the world. However, although yogurt consumption is good for human health, as reported [15], does the influence of the yogurt consumption on human gut microbiome also go against gut resistome? This study therefore focuses on deciphering the composition changes among healthy humans in comparison with antibiotic usage and yogurt consumption.

## 2. Results

### 2.1. Baseline Characteristics among Study Groups

The average age of the three groups ranged from 48.0 to 54.7 years, with a slightly higher proportion of women than men. The yogurt group had higher levels of education and lower rates of smoking than the other two groups. Other basic characteristics, such as body mass index (BMI), waist circumference (WC), physical activity (PA), and total energy were presented as means in Table 1.

### 2.2. Composition of the Gut Microbiota in groups of Antibiotic Usage and Yogurt Consumption

**Figure 1:**
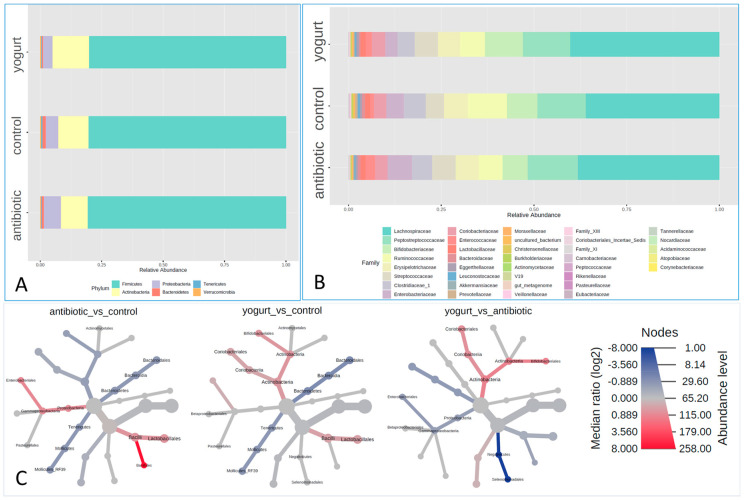
Composition of the Gut Microbiota of control, antibiotic usage and Yogurt consumption groups. (**A**) Gut microbiome composition at the phylum level; (**B**) Gut microbiome composition at the family level; (**C**) Heat tree demonstration of gut microbiome composition at the order level.

In our study, bacterial 16S rRNA sequences from 1113 healthy human fecal samples were identified. The composition of gut microbiota was shown in phylum and family levels, respectively (Figure 1A,B). The relative abundances of the *Bacteroidetes* phyla in feces from the antibiotic usage and yogurt group were both lower than those of the control group, with the yogurt group being the lowest among them. The relative abundances of *Actinobacteria* and *Proteobacteria* phyla showed opposite results while comparing with the control group, with *Actinobacteria* higher in the yogurt group, *Proteobacteria* higher in the antibiotic group, and control in the middle. At the order level, the relative abundances of *Bacillales* order in the antibiotic usage group increased notably. Both antibiotic and yogurt brought about observable increases in *Lactobacillales* order (Figure 1C). At the family level, the relative abundances of *Streptococcaceae, Enterobacteriaceae* and *Enterococcaceae* families in antibiotic users increased almost 50%, 70% and 200%, respectively, when compared with the control. Interestingly, the yogurt consumption group was also composed of increased relative abundances of *Streptococcaceae* and *Enterococcaceae* family, but not the *Enterobacteriaceae* family, compared with control (Figure 1B). 

### 2.3. Alpha and Beta Diversity in the Gut Microbiome among Antibiotic Usage, Yogurt Consumption and Control

**Figure 2:**
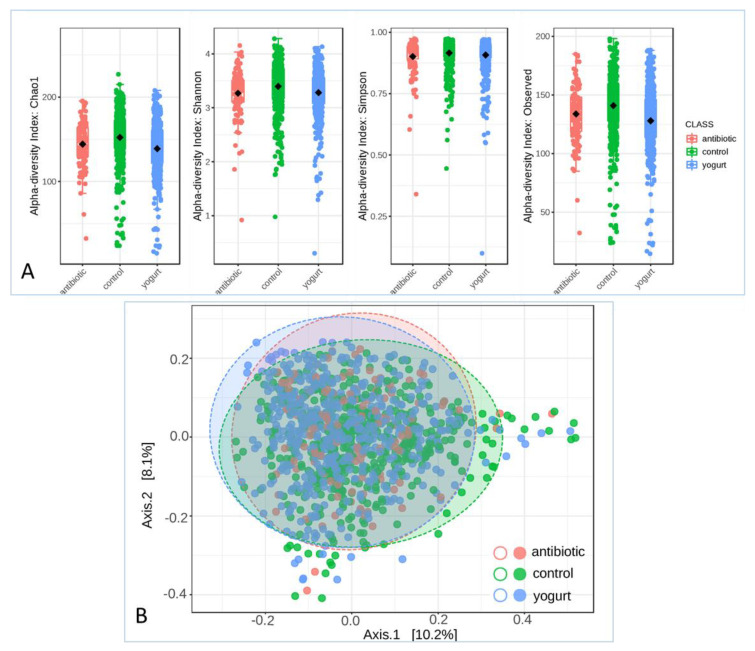
Alpha and Beta Diversity of the Gut Microbiome. (**A**) Alpha diversity evaluation at Chao1, Shannon, Simpson and observed indexes; (**B**) Beta diversity based on the PERMANOVA statistic method.

After operational taxonomic units (OTUs) were obtained and analyzed using QIIME 2 work flow, alpha and beta diversity analyses were carried out. Chao1, Shannon, Simpson and observed indexes were calculated to evaluate the alpha diversity in the human gut microbiome. The microbiomes of the antibiotic usage and yogurt consumption groups exhibited a lower alpha diversity than that of the control (Figure 2A). There was a significant difference in the Chao1, observed OTUs, and Shannon indices (*p* = 1.0796 × 10^−10^, *p* = 1.7389 × 10^−12^, and *p* = 0.0001, respectively). Principal coordinates analysis (PCoA) was performed to visualize the beta diversity based on the PERMANOVA statistic method in the microbial community structure, where antibiotic usage and yogurt consumption groups were shown in the Bray–Curtis Index analysis (Figure 2B, F-value: 8.3963; R-squared: 0.014916; *p*-value: 0.001).

### 2.4. Biomarker Differences among Antibiotic Usage, Yogurt Consumption and Control

**Figure 3:**
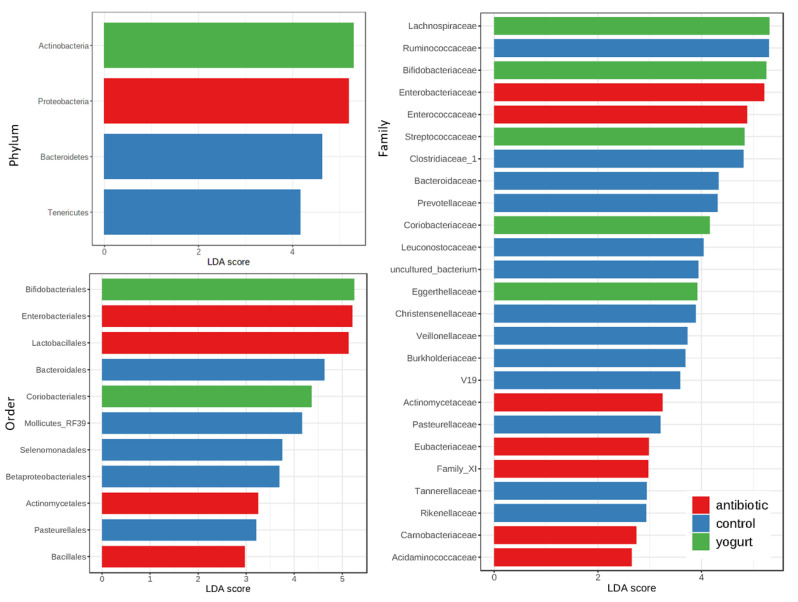
Biomarkers of different taxa level of the Gut Microbiome among antibiotic usage, Yogurt consumption and control (LEfSe analysis).

In this study, we carried out the linear discriminant analysis (LDA) effect size (LEfSe analysis) to investigate the differences in different taxa levels between groups using the Microbiome Analyst online platform [16,17]. At phylum level, *Proteobacteria* was the biomarker of the antibiotic usage group, and *Actinobacteria* the yogurt consumption group, where they were all four times higher than those in the control group. At Order level, *Enterobacteriales, Lactobacillales,* and *Actinomycetales* in the antibiotic usage group were three times higher than those in the control group. At family level, the number of biomarkers in the yogurt consumption and antibiotic usage group were respectively 5 and 7, lower than that of the control (13), as shown in Figure 3.

## 3. Discussion

Self-use of antibiotics in healthy humans is becoming a health concern, especially in low-income groups of people. Although clinical use of antibiotics has been proved to reduce diversity in the gut microbiome [8], the situation for antibiotic usage in healthy humans is unclear. As an important part of healthy human diet, how yogurt consumption influences human gut microbiome is under researched. Therefore, in order to better understand the effects of antibiotic usage and yogurt consumption on human gut, it is important to understand the similarities and differences between antibiotic usage and yogurt consumption among healthy humans. 

Both the antibiotic usage and yogurt consumption groups exhibited a decrease in the richness and evenness of diversity compared with the control group. The insignificant result of the Simpson index showed that the dominance indices of gut flora remained stable in a large scale of healthy human gut microbiome. Alpha analysis indicated that antibiotic usage and yogurt consumption were making significant changes in healthy human gut microbiome. However, the overall diversity of the microbiome among these groups was not obvious enough, possibly due to attenuation of the potential effects on the physiology of the host organisms. This may also possibly be due to the limitation of the sample sets, and the deviation caused by other variables such as age, smoking state, and the evolutionary outcomes that keep getting the human gut microbiome back to a stable state should also be considered.

Yogurt is produced by adding a combination of probiotics to ferment milk, which are mostly *Lactobacillus delbrueckii subspecies bulgaricus* and *Streptococcus salivarius subspecies thermophiles*, *Lactobacilli* or *Bifidobacteria* [18]. The lactic-acid-secreting bacteria that are added to milk may modify the intestinal environment in two ways: (1) increasing tight junctions in the gut epithelium; (2) decreasing potentially harmful enzymes produced by the residential bacteria [19,20]. In our study, the relative abundances of the *Bacteroidetes* phyla in feces from the antibiotic usage and yogurt groups were both lower than those of the control group, with the yogurt group being the lowest among them. It is consistent with Odamaki’s reports that the consumption of yogurt containing *Bifidobacterium longum* BB536 significantly decreases enterotoxigenic *Bacteroides fragilis* in the gut microbiota [21].

Yogurt consumption was reported to be good for human health, and is especially deemed beneficial to the human gut [22]. Yogurts made with *Bifidobacterium lactis* and other probiotics are considered to help maintain gut flora by providing organisms that are usually inhabited in the human gut [23]. However, we need to be careful when antimicrobial resistance is taken into consideration. It has been reported that antibiotic usage would assist AMR genes’ horizontal transfer in patients [10]. However, the mechanisms of antibiotic resistance transmission during microbiome modification remain unclear. At the family level of the relative abundances in this study, *Streptococcaceae*, *Enterobacteriaceae* and *Enterococcaceae*, which are closely correlated with AMR, increased almost 50%, 70% and 200% in the antibiotic usage group, as expected. It has been suggested that probiotic supplementation may decrease the total load of ARGs within the gut [24,25]. However, in our study, interestingly, the yogurt consumption group was also composed of increased relative abundances of *Streptococcaceae* and *Enterococcaceae* family, the same as antibiotic usage group. Therefore, it is not negligible that we should consider the potentiality of yogurt consumption on bacterial ecosystems that will potentially increase the stress and/or selection pressure and, therefore, could induce an intensification of ARG gene transfer processes from commensal bacteria to pathogens in the human gut, posing a possible risk for human health.

Through LEfSe analysis, we can see that the number of biomarkers in the yogurt consumption and antibiotic usage group was much lower than that of control. This probably means that the yogurt consumption and antibiotic usage decreased gut micobiome diversity in healthy human. Antibiotic usage was more influential than yogurt consumption on healthy human gut microbiome. However, antibiotic usage and yogurt consumption do share more identical changes in healthy human gut microbiome than disparities. Therefore, in order to reduce the health and AMR transmitting risk, functional research based on an in-depth study from a meta-interactomics perspective and the use of advanced computing equipment under different metabolic states needs to be carried out to decipher the correlation between antibiotic usage and yogurt consumption on human gut microbiota.

## 4. Materials and Methods

### 4.1. Study Design and Participants

The present study was based on data from the China Health and Nutrition Survey (CHNS), an ongoing, population-based longitudinal cohort in China that covers lifestyle, diet and disease status, physical measurements and biological indicators. A total of 15 provinces/megacities in China participated. An overview of the CHNS study design has been published previously [26]. During the 2015 survey, stool samples were collected as well as dietary information. In the study, 16S rRNA analysis from stool samples was used to construct gut microbiota profiles (n = 3248). Participants were excluded if they had no FFQ information during 2015 (n = 9), or drank more than 150 g of yogurt more than once a week and took antibiotics within 6 months at the same time (n = 25). Participants were included if they drank yogurt more than once a week and consumed more than 150 g (n = 497, yogurt group), or had not drunk yogurt (n = 1987) in the past year, or had taken antibiotics within 6 months (n = 122, antibiotic group). The non-yogurt-drinkers were matched with the yogurt group 1:1 for gender and age (no more than 2 years’ difference), and 494 people were finally matched as the control group (n = 494, control group). A total of 1113 participants from the 2015 survey were included in the present study (age 48.9 ± 13.5 years, mean ± SD). 

### 4.2. Sample Collection

Adult participants collected stool samples themselves after receiving adequate instruction for the collection process during a home visit prior to collection, and samples were frozed immediately at −20 °C. Stool samples were transported within 48 h by cold chain to the central laboratory and stored at −20 °C to ensure proper processing.

### 4.3. Genomic DNA Extraction

The methods for DNA extraction, amplification and sequencing have been described previously [27]. A bead-beating procedure was used to extract bacterial DNA (TIANGEN Biotech, Beijing, China) following the manufacturer’s instructions. For 16s ribosomal RNA (rRNA) genes, we adjusted the DNA concentration of each sample to 50 ng/L.

### 4.4. PCR Amplification of the V3-V4 Region of 16S rRNA Gene

The V3-V4 region of 16s rRNA gene with a 6-bp barcode unique to each sample was amplified with primers 515F/806R (5′-GTGCCAGCMGCCGCGGTAA-3′/5′-GGACTACHVGGGTWTCTAAT-3′) to characterize the taxonomic profile of gut microbiota. In an equimolar ratio, PCR products were combined. An Illumina HiSeq PE-250 platform was used to sequence the libraries, constructed with TruSeq DNA PCR-Free Library Preparation Kit (Illumina, CA, USA). 

### 4.5. Microbial Data Analyses

The comparisons between groups were analyzed using parametric (chi-square test, analysis of variance) or non-parametric tests (Kruskal–Wallis test); a *p*-value was assessed as significant when <0.05. 

An analysis of the 16S rRNA gene sequences was performed using the QIIME 2 bioinformatics pipeline [28]. The filtering and normalization, visualization of the data, alpha diversity, beta diversity, heat tree and LEfSe analysis were all produced using a web-based platform Microbiome Analyst [16,17]. The parameters for data filtering were minimum count = 4, prevalence in samples = 20%, percentage to remove based on inter-quantile range = 10%, sample size = 5000.

## 5. Conclusions

Antibiotic usage and yogurt consumption demonstrated significant changes in specific bacterial groups (*Streptococcaceae*, *Enterococcaceae* and so on) in healthy human gut microbiomes in this study. Antibiotic usage and yogurt consumption shared more identical changes in healthy human gut microbiome than disparities, especially ARG gene related bacteria groups that could induce an intensification of ARG gene transfer processes from commensal bacteria to pathogens in human gut. However, in order to understand the potential risks of antibiotic usage and yogurt consumption on antibiotic resistance transmission in human gut microbiota, further researches need to be carried out.

## Figures and Tables

**Table 1:** Baseline characteristics of adults in different groups.

Characteristics	Control Group (n = 494)	Yogurt Group (n = 497)	Antibiotic Group (n = 122)
Gender (n, %)			
Male	202 (40.9)	202 (40.6)	58 (47.5)
Female	292 (59.1)	295 (59.4)	64 (52.5)
Age (year)	48.5	48.0	54.7
BMI (kg/m^2^)	25.0	23.9	24.5
WC (cm)	86.3	83.4	85.7
Smoke (n, %)			
No	372 (75.6)	402 (81.1)	87 (71.9)
Yes	120 (24.4)	94 (18.9)	34 (28.1)
PA (METs/week)	148.6	117.0	140.7
Education (n, %)			
Primary and below	104 (23.5)	36 (7.7)	21 (21.6)
Junior high	175 (39.6)	136 (29.0)	42 (43.3)
Senior high and above	163 (36.9)	297 (63.3)	34 (35.1)
Total energy (kcal/d)	2000.0	2043.1	1931.9

## Data Availability

Data are available upon reasonable request.
